# The Impact of Armed Conflict on the Epidemiological Situation of COVID-19 in Libya, Syria and Yemen

**DOI:** 10.3389/fpubh.2021.667364

**Published:** 2021-06-11

**Authors:** Mohamed A. Daw

**Affiliations:** Department of Medical Microbiology and Immunology, Faculty of Medicine, University of Tripoli, Tripoli, Libya

**Keywords:** Libya, Syria, Yemen, epidemiology, corona

## Abstract

**Background:** Since the Arab uprising in 2011, Libya, Syria and Yemen have gone through major internal armed conflicts. This resulted in large numbers of deaths, injuries, and population displacements, with collapse of the healthcare systems. Furthermore, the situation was complicated by the emergence of COVID-19 as a global pandemic, which made the populations of these countries struggle under unusual conditions to deal with both the pandemic and the ongoing wars. This study aimed to determine the impact of the armed conflicts on the epidemiology of the novel coronavirus (SARS-CoV-2) within these war-torn countries and highlight the strategies needed to combat the spread of the pandemic and its consequences.

**Methods:** Official and public data concerning the dynamics of the armed conflicts and the spread of SARS-COV-2 in Libya, Syria and Yemen were collected from all available sources, starting from the emergence of COVID-19 in each country until the end of December 2020. Datasets were analyzed by a set of statistical techniques and the weekly resolved data were used to probe the link between the intensity levels of the conflict and the prevalence of COVID-19.

**Results:** The data indicated that there was an increase in the intensity of the violence at an early stage from March to August 2020, when it approximately doubled in the three countries, particularly in Libya. During that period, few cases of COVID-19 were reported, ranging from 5 to 53 cases/day. From September to December 2020, a significant decline in the intensity of the armed conflicts was accompanied by steep upsurges in the rate of COVID-19 cases, which reached up to 500 cases/day. The accumulative cases vary from one country to another during the armed conflict. The highest cumulative number of cases were reported in Libya, Syria and Yemen.

**Conclusions:** Our analysis demonstrates that the armed conflict provided an opportunity for SARS-CoV-2 to spread. The early weeks of the pandemic coincided with the most intense period of the armed conflicts, and few cases were officially reported. This indicates undercounting and hidden spread during the early stage of the pandemic. The pandemic then spread dramatically as the armed conflict declined, reaching its greatest spread by December 2020. Full-blown transmission of the COVID-19 pandemic in these countries is expected. Therefore, urgent national and international strategies should be implemented to combat the pandemic and its consequences.

## Introduction

Armed conflicts have major effects and grave consequences that are difficult to deal with. Historically, wars disrupted the human-microbe balance, resulting in devastating outbreaks of microbial diseases and high rates of mortality and morbidity all over the world (1). In the beginning of the last century, the spread of *Yersinia pestis* (the causative agent of plague) was aggravated by fleeing from war zones, which increased the geographical range of the epidemic (2). During the global spread of COVID-19, the technically advanced nations focused on the domestic impact of COVID-19 just as the disease is likely spreading intensely in poor and war-affected countries, where it can wreak havoc in these fragile states. This is evident in Libya, Syria and Yemen, which have been locked in destructive armed conflict for almost a decade now (3). These conflicts have resulted in widespread death, injury and population displacement, as well as serious destruction of health care system, which made them ill-prepared for COVID-19 (4).

The oil-rich country of Libya was plunged into chaos after a 2011, NATO-backed uprising toppled and killed the long-standing socialist leader Muammar Gaddafi and split the country into two rival governments (5). In Syria, the first phase of the conflict was ignited by protests in early 2011, which were dubbed as the Arab Spring uprisings. The situation was further complicated by international sanctions and the military intervention of foreign powers, including Russia, USA, Europe, Turkey, the Arab Gulf States, and Iran (6).

In Yemen, the conflict started after 2011, but since then the country went through a series of political upheavals resulting in a civil war complicated by international intervention lead by Saudi-Arabia and the United Arab Emirates in 2015. Consequently, nearly 100,000 people have died, 250,000 have been displaced, and 80% of the population are in need of assistance and protection. These added further complexity to the COVID-19 pandemic and hampered control measures (7).

The healthcare systems of these three countries, which are on the brink of collapse, can hardly mitigate the spread of epidemics. They were the last countries in the Middle East and North Africa region to report their first COVID-19 infections. In Syria, the country reported the first case on March 22nd 2020, followed by Libya on March 24th, and then Yemen on April 10th (8). These countries are not prepared to confront the pandemic alone (9). Furthermore, ongoing conflict can hinder the efforts to fight the pandemic and may act as a catalyst for its spread; more serious consequences are expected. However, the effect of armed conflict on the dynamics of COVID-19 is multifaceted. The objectives of this study were to analyze the effects of the conflict on the epidemiological situations of the pandemic in Libya, Syria and Yemen, and to outline the strategies needed to combat COVID-19 and its consequences in these violent regions.

## Materials and Methods

### Data Collection

#### COVID-19 Dataset

All official and public data concerning the dynamics of the armed conflict and of COVID-19 in Libya, Syria and Yemen were collected from the onset of the COVID-19 pandemic till the end of December 2020. They included the total number of confirmed infections, deaths, and recoveries. These data were collected from the official data of the Libyan Ministry of Health and the daily reports on the Facebook page of the National Center for Disease Control (https://ncdc.org.ly/Ar/).

For Syria, the data were obtained from the Ministry of Health daily and the COVID-19 updates on its Facebook page (https://www.facebook.com/MinistryOfHealthSYR), as well as from the Worldometers international counter (https://www.worldometers.info/coronavirus). For Yemen, the data were collected from Worldometers (https://www.worldometers.info/coronavirus/). Publicly available mobility data were collected from Google (https://www.google.com/covid19/mobility/), which provides data on movement in each country and from the Government Response Tracker of Oxford Covid-19 (https://www.bsg.ox.ac.uk/research/research-projects/covid-19-government-response-tracker), which provides real-time data on the pandemic spread worldwide (10). Besides, we used the information and data related to COVD-19 operated by the Johns Hopkins University Center for Systems Science and Engineering (JHU CSSE) to determine the upsurge in the hostilities and how that affected the prevalence of COVID-19 dynamics Libya, Syria and Yemen (11).

#### Armed Conflict Dataset

Armed conflict encompasses all forms of wars and events that cause population death and displacement, including civil wars, insurgencies, rebellions, and battles. Each conflict was sorted by date and duration. Furthermore, armed conflict events in Libya, Syria and Yemen were selected from the Armed Conflict Location & Event Data Project (ACLED) (https://www.acleddata.com). This project reports the most reliable and accurate data on all types of violence, including armed conflict and political unrest and protests, including the place, time, fighting groups, and political parties involved, particularly in Asia, Africa, Latin America, and Eastern Europe (12).

### Statistical Analysis

Data analysis was carried out by using Xl-Stat2017 and PAST, version 2.17c. A simple smoother was first applied on a weekly basis to assess peaks and trends of the armed conflicts and of COVID-19 spread (cumulative number of cases and deaths) (13).

## Results

The armed conflict and the epidemiological manifestations of COVID-19 in Libya, Syria and Yemen have gone through various stages and different scenarios. Based on the available data, the trajectory of conflicts and the emergence of the global pandemic by March 2020 vary greatly among the three countries.

### The Case of Libya

**Figure 1:**
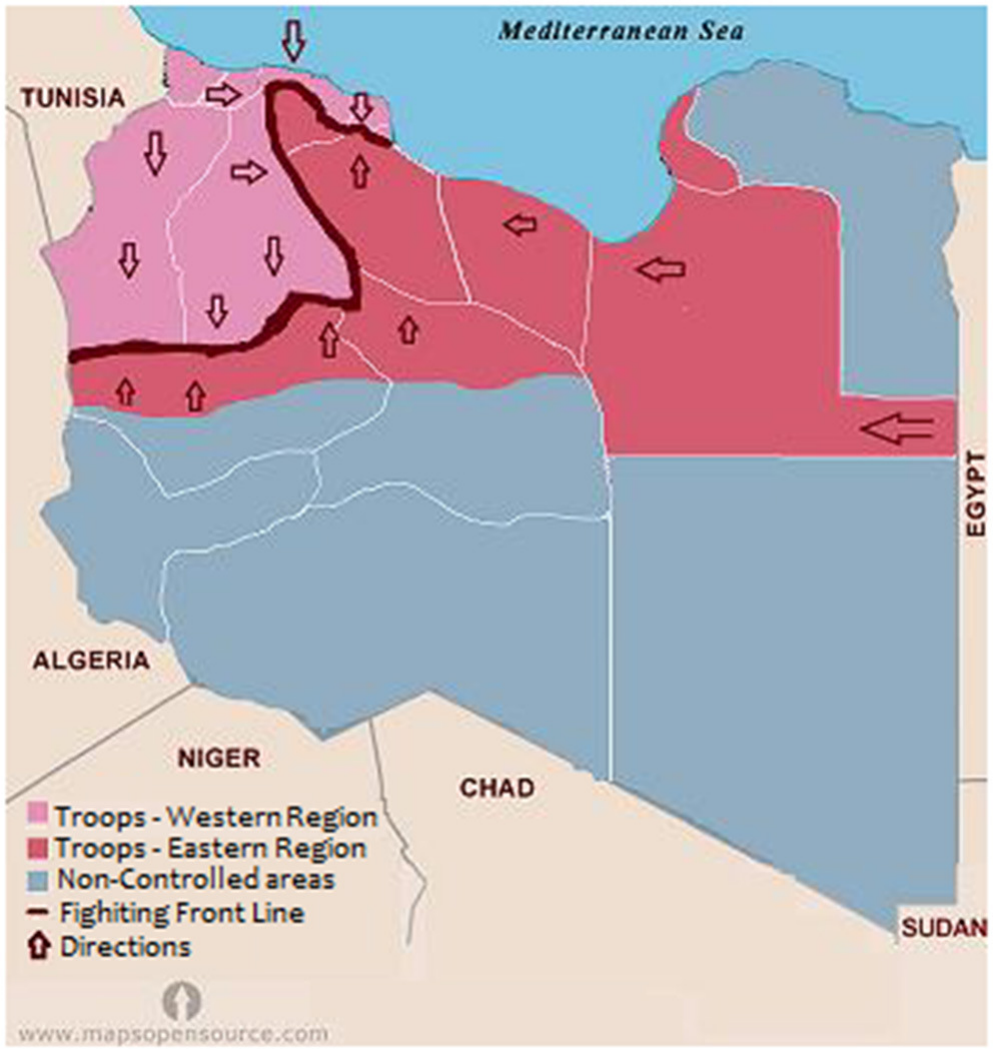
Map of Libya showing the geographic areas controlled by each fighting group during the emergence of COVID-19 in 2020.

The Libyan armed conflict started in 2011, and with the intervention of NATO, the long-standing socialist regime was toppled. Three years later, the country has passed through different upheavals that resulted in the country being split between two rival governments, one in the east controlling most of the Libyan territory and the other in the west. Figure 1 shows the geographic area controlled by the fighting groups. Tripoli and the central area were controlled by the Tripoli authority, while the eastern region, Sebha and most of the western mountain regions were under the control of the eastern government. The southern border region was under the control of other militias.

This conflict culminated in April 2019 with an assault on the capital, Tripoli, in the west by the forces of the eastern region. This assault has caused over 20,000 deaths, displaced more than 210,000 others. The siege of Tripoli was rebuffed in August 2020, and subsequent local and international efforts succeeded in re-establishment of a unified government in March 2021. As an overall consequence of the armed conflicts in Libya, 1.3 million people require humanitarian assistance.

**Figure 2:**
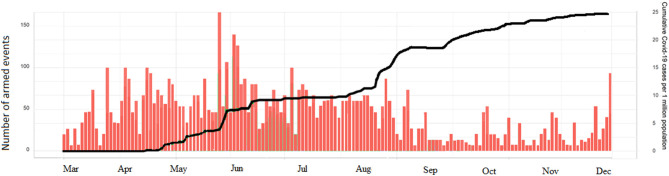
Intensity of the armed conflict and patterns COVID-19 cases in Libya during the epidemic period (March until December 2020).

**Figure 3:**
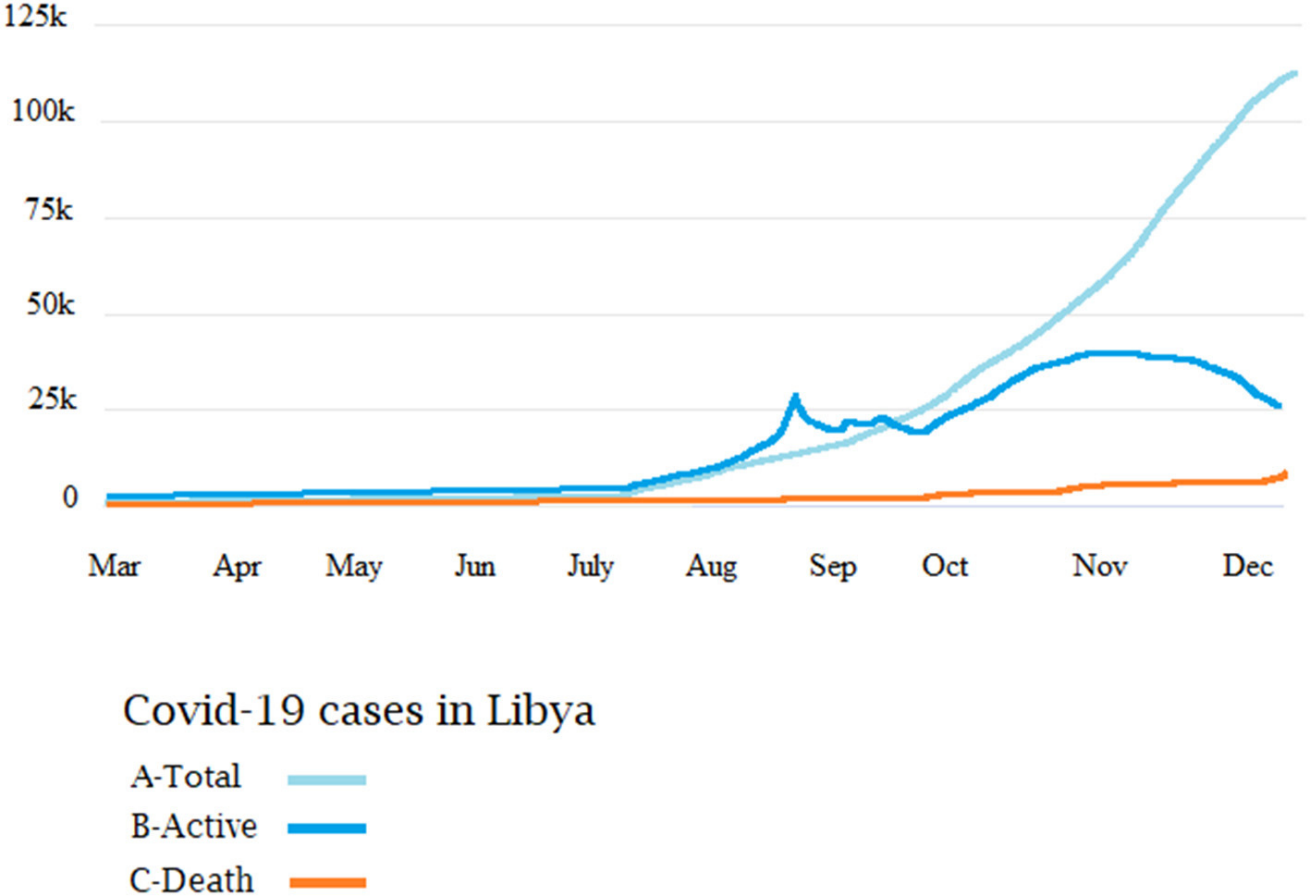
The epidemiological patterns of total cumulative cases **(A)**, active cases **(B)**, and deaths **(C)** of COVID-19 in Libya during 2020.

Figure 2 shows the correlation between the intensity of the armed conflict and the emergence of COVID-19 pandemic in Libya. In March 2020, few cases of COVID-19 were reported in the first eight epi-weeks. Then the reported cases increased significantly to reach over 500 cases/day by August 2020. By December 2020 (epi-week 51), a total of 95,708 infections had been reported (Figure 3), of which 28,247 were still infected, 66 076 had recovered and 1,385 had died. These figures translate into 1,405 cases of COVID-19 per 100,000 population and 20 deaths per 100,000 population, giving a national case fatality rate of 1.4%. The largest number of cases per 100,000 population were in Tripoli (3,874), Misrata (2,061), and Jabal al Gharbi (1,515), followed by Sebha (935/100,000) and Benghazi (367/100,000). Mortality was high in Al Kufra (76/100,000), Nalut (52/100,000), Zwara (43/100,000), and Azzawya (42/100,000).

### The Case of Syria

**Figure 4:**
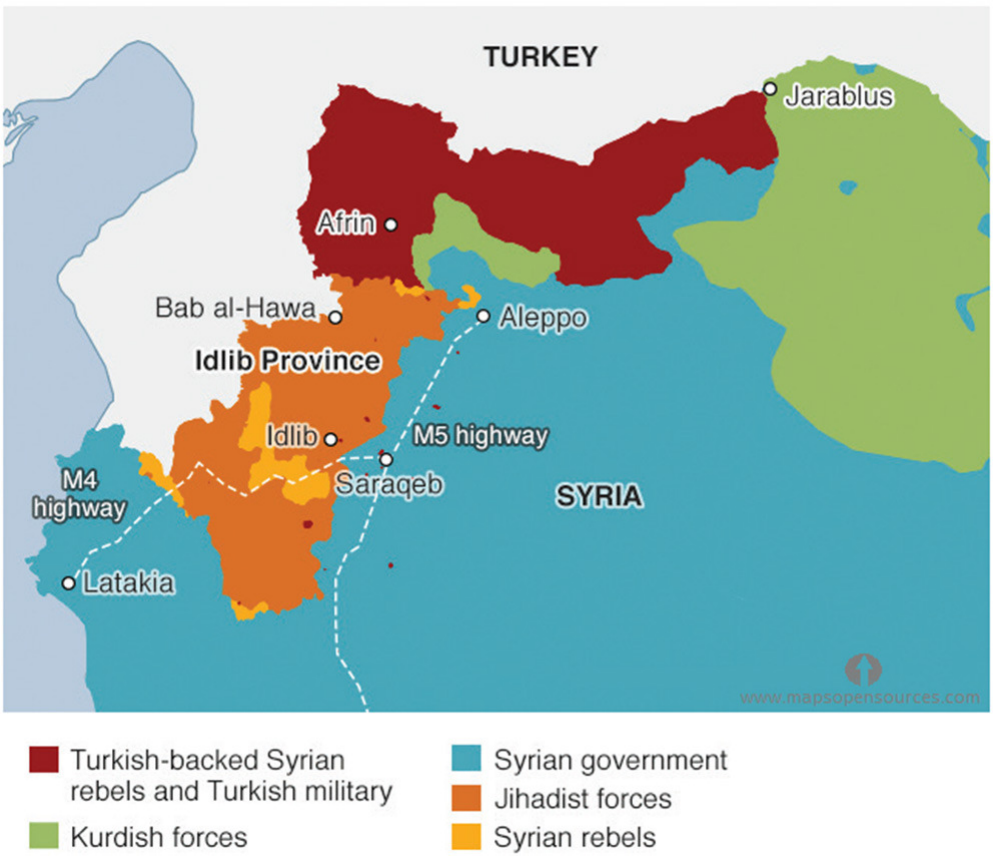
Map of Syria showing geographic areas controlled by each armed group during 2020.

For over 10 years, Syria has been plagued by war that has torn the country into five different areas and fragmented it into many areas controlled by militias and fighting groups (Figure 4). Moreover, it destroyed the national healthcare system. The armed opposition groups still control large parts of the northern regions, including Jarablus, Afrin, Idlib Province, and Hayat Tahrir al-Sham, while the Syrian government has regained control of most of the other parts of the country.

**Figure 5:**
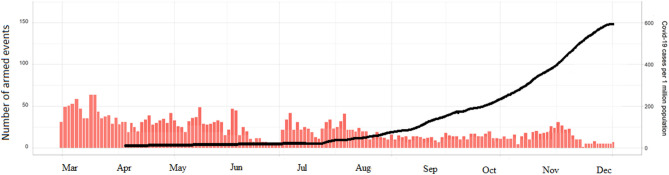
The levels of armed violence in Syria and the spread of COVID-19 during 2020.

In 2020, the Syrian armed conflict was intense, particularly in Hayat Tahrir al-Sham and Idlib, at the time as the arrival of COVID-19 (Figure 5). The level of violence in Syria remains high and attacks on healthcare facilities also appear to have increased. The first case of COVID-19 in Syria was diagnosed on March 23, 2020. Thereafter, for the first 2 months, the virus spread slowly, never infecting more than ten people per day. By July, the number of reported cases started to increase and reached its highest by December 2020.

**Figure 6:**
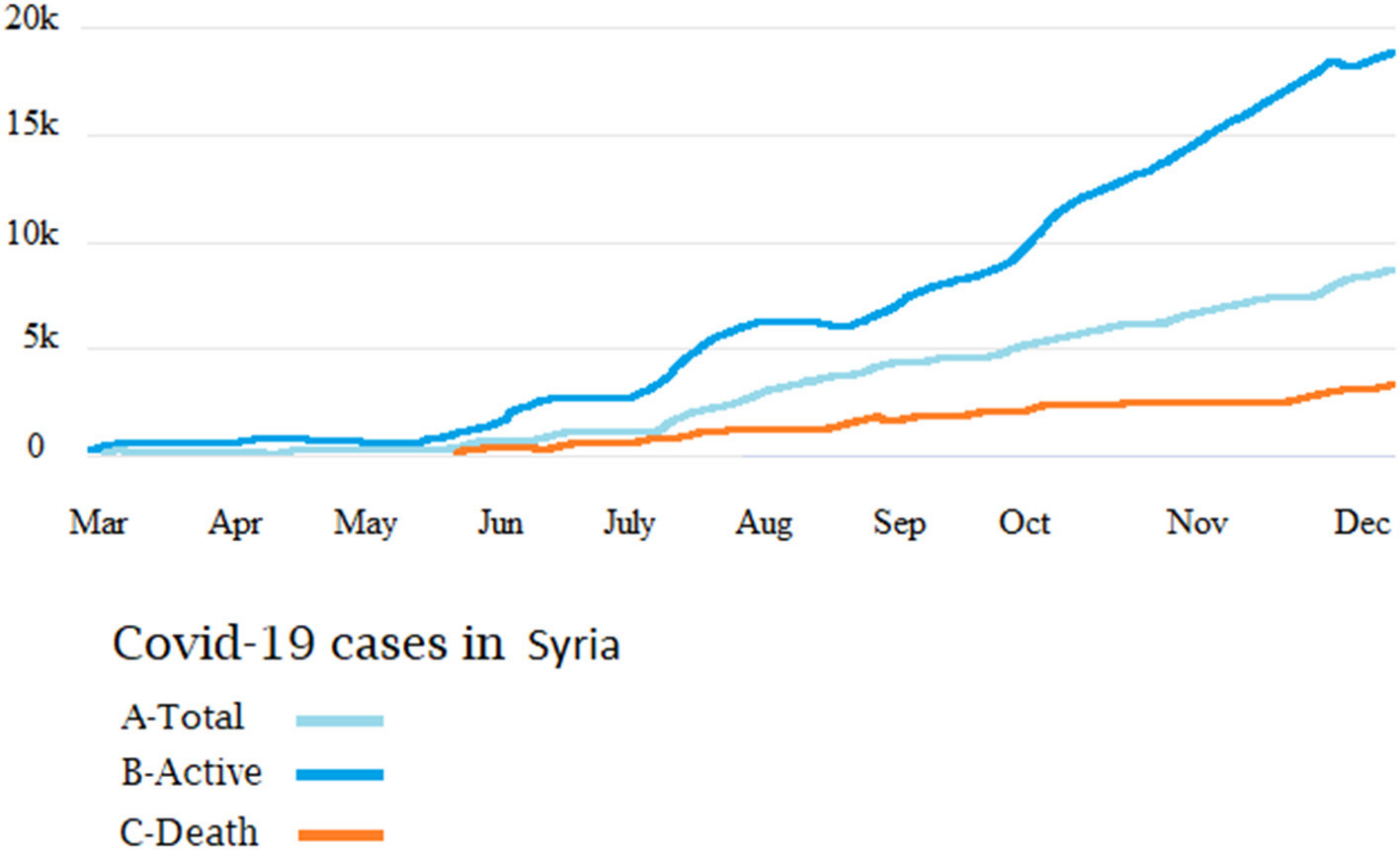
The number of officially reported cases of COVID-19 in Syria. Cumulative cases **(A)**, recoveries **(B)**, and number of deaths **(C)**.

Figure 6 shows the accumulative cases of COVID-19 in Syria. A total of 13,885 corona viruses cases were reported. The prevalence was steady till July 18 (epi-week 16), after which a slight increase was noticed from August 1st till October 24th. A sudden increase was noticed from November 7th till December, when the reported number of cases was highest. During this period, 7,329 patients recovered and 906 deaths were officially reported. However, the estimated number of deaths may reach 4,340 (95% CI: 3,250–5,540). The highest estimated number of new infections, including both asymptomatic and symptomatic cases, was in Damascus (9,760; 95% CI: 6,470–11,360). No COVID-19 cases have been officially confirmed yet from Idlib Jarablus, Afrin, and Hayat Tahrir al-Sham.

### The Case of Yemen

**Figure 7:**
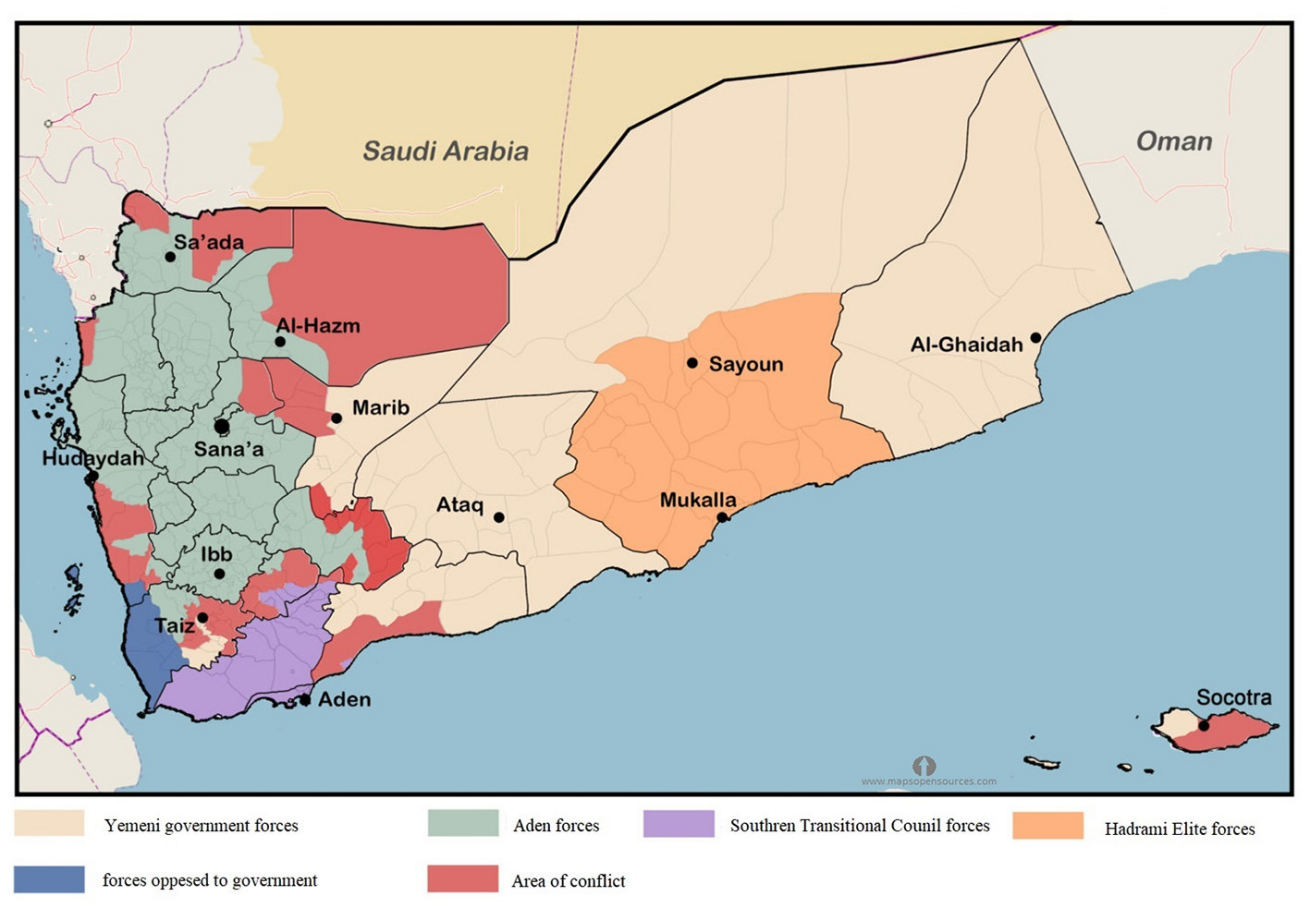
Map of Yemen showing the zones of the armed conflict and areas controlled by each fighting group during 2020.

After Yemen's 2011 uprising, the country has been divided into five counties with different military and political authorities (Figure 7). These include northern counties controlled by Huthi authority and areas controlled by the Eden government, including Norther Hadramawt, Marib, ak-Mahra, aljawf, Shebwa, Tazz City, and Abyan.

**Figure 8:**
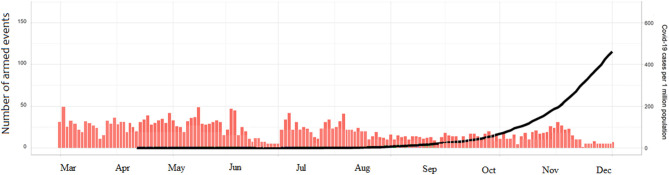
Levels of violence and COVID-19 cases in Yemen during 2020.

On April 24, 2020, intense military activity restarted between the Aden authority and the forces in the southern region. This escalation was in parallel with the spread of the COVID-19 pandemic within the country (Figure 8). Yemen reported the first case of COVID-19 on April 10th were full, 2020. Since then, The Eden authority reported 323 cases including 80 deaths, while Sana reported only four cases and one death.

**Figure 9:**
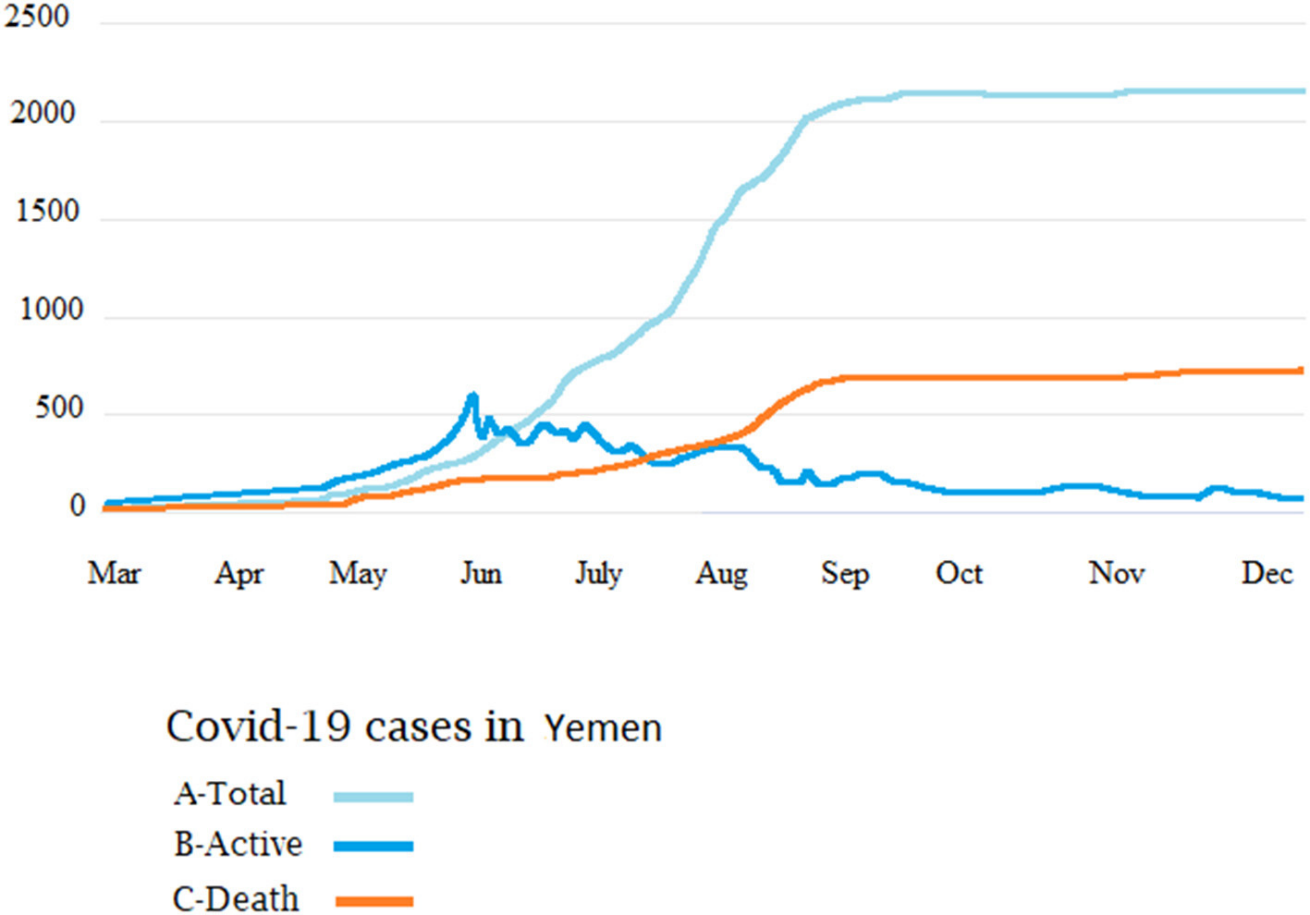
Cumulative COVID-19 cases in Yemen. Total cases **(A)**, recoveries **(B)**, and number of deaths **(C)**.

Figure 9 shows a total of 2,120 cases with 1,425 recoveries by December 2020. The rate of new infections started to increase substantially at the end of April, and by August 11th (epi-week-12) the country had 1,832 confirmed cases. By the 26th of September (epi-week 16), the reported cases reached up to 2,034. The number increased substantially by December 31, by which time there were 615 deaths. Most of these cases were reported by the Aden-controlled government.

## Discussion

Since its emergence, COVID-19 has affected the whole world. Most epidemiological studies have been carried out in developed countries and in politically stable developing countries. Studies on the effects of war on the spread of COVID-19 in conflict hotspots were rarely reported. The effect of armed conflict on the prevalence of infectious diseases is multi-faceted and complex (12). This is clearly evident in Africa during the spread of Ebola (14). However, contradictory observations of both increases and decreases in the spread of infectious disease during and after conflicts have been noticed.

Libya, Syria and Yemen have experienced three of the most devastating armed conflicts in recent history. Over 10 years of ongoing armed conflict have resulted in thousands of people being injured, killed and displaced, with major destruction of hospitals and other healthcare services. In Libya, about 1.3 million citizens require humanitarian aid, 200,000 have been displaced, and there are about 636,000 migrants and refugees (5, 13). In Syria, half of the population are in urgent need of aid, over six million have been displaced, and 83% live below the poverty line. The situation is even worse in Yemen, as the country has been experiencing one of the most devastating crises in human history since World War II, and most of the population need aid in order to survive (15). These situations have resulted in a hidden spread of the pandemic in these three countries (16). There is an urgent need for quantitative assessment of the effects of the conflict on the spread of the pandemic in war-torn countries.

These three countries were the last countries to report the first cases of COVID-19 in the region. Syria's first case was reported on March 10th 2020, followed by Libya on March 24th and Yemen on April 10th (7). At the start of the pandemic, only few COVID-19 cases were reported in each country and then the numbers increased steadily. The armed conflict became even worse during the pandemic spread, particularly in Libya (17).

In Libya, only 156 cases were reported in the early months of the epidemic (March–July) and then increased drastically up to 1,000 cases/day by August-December. This accompanied escalation of the armed conflict from April 4, until August 2020. Similar patterns were noticed in Syria and Yemen, though the number of confirmed cases in these two countries was less than that in Libya. By December 21st 2020 (epi-week 51), Libya reported the highest number of COVID-19 cases, it was listed as number 10 in the MENA region, and it was in the 6th place in the number of deaths (18, 19). Similar results were reported in Syria and Yemen. A modeling study carried out by Watson et al. with the assumption that the epidemic had passed its peak transmission, estimated that the burden of COVID-19 in Syria was immense and that a total of 39.0% (95% CI: 32.5–45.0%) of the population had been infected by 2nd September 2020 (20). Of the three conflicts, Yemen faces the worst COVID-19 outlook, and the actual number of cases is likely much higher than the reported numbers. By August, Yemen reported 1,832 confirmed cases and 518 deaths. This death rate is five times higher than the world average (21).

Our data indicate that the armed conflict in these countries has masked the actual status of the epidemic at an early stage. This is in concordance with previous data published by Daw et al. which showed that the ongoing armed conflict in these volatile regions has influenced the spread of the pandemic either by masking the actual prevalence in the controlled areas or accelerating its spread in the non-controlled regions (22).

The data collected from these war-torn countries were undercounts and do not represent the real situation. This has been due not only due to poor response from the authorities but also to apparent concealment of cases, particularly in Sana-Yemen, northern Syria, and eastern Libya. Furthermore, lack of testing, population displacement, malnutrition, and poverty are the main factors that hamper obtaining accurate data on the epidemiological situation in Libya, Syria and Yemen.

This study illustrates the impact of the conflicts on the spread of the pandemic in these war-torn countries. However, certain limitations of the study have to be mentioned. First, the data were obtained partly from gray literature, which has implications. Second, the data were neither standardized nor validated and should therefore be interpreted with caution (23, 24). Furthermore, diagnostic capacity in each country was difficult to evaluate and no accurate data were collected regarding the number diagnostic tests carried during the armed conflict period. Hence then, intervention programs should be based on reality that full-blown spread is evident within these countries. Therefore, urgent national and international strategies should be implemented to combat the pandemic and its upcoming consequences (25, 26).

## Conclusion

This study is one of the first studies to analyze the impacts of armed conflicts on the epidemiological manifestations of the COVID-19 pandemic. Based on our analysis, evidence is emerging that there was a surge and intensification of armed conflict in Libya, Syria and Yemen, particularly in the first 4–6 months of the spread of the COVID-19 pandemic. This resulted in a hidden spread of the pandemic during the early stage, which was exacerbated lately, resulting in a high number of infected cases, suggesting a synergistic interaction between the COVID-19 pandemic and the ongoing conflict in these countries. This indicates that the pandemic and its effects are likely to evolve for years if not dealt with (27, 28). Healthcare systems in these countries face serious challenges, as SARS-CoV-2 could spread rapidly through the populations, particularly among those in the most vulnerable groups, including injured patients, internally displaced people, prisoners, and immigrants. Hence, global interventions are needed to stop the armed conflicts (as happened in Libya), protect health workers and health facilities, and provide sufficient humanitarian support to prepare for an impending crisis (29, 30).

## Data Availability Statement

The original contributions presented in the study are included in the article/supplementary material, further inquiries can be directed to the corresponding authors.

## Author Contributions

MD is sole author who conceived, designed the study, collected and analyzed the data, wrote and revised the manuscript, and approved the final manuscript.

## Conflict of Interest

The author declares that the research was conducted in the absence of any commercial or financial relationships that could be construed as a potential conflict of interest.
